# Factors Associated with Beta-Cell Dysfunction in Type 2 Diabetes: The BETADECLINE Study

**DOI:** 10.1371/journal.pone.0109702

**Published:** 2014-10-27

**Authors:** Giuseppina T. Russo, Carlo Bruno Giorda, Stefania Cercone, Antonio Nicolucci, Domenico Cucinotta, on behalf of BetaDecline Study Group

**Affiliations:** 1 Department of Clinical and Experimental Medicine, University of Messina, Messina, Italy; 2 Metabolism and Diabetes Unit ASL TO5, Chieri, Italy; 3 MSD, Rome, Italy; 4 Department of Clinical Pharmacology and Epidemiology Fondazione Mario Negri Sud, S. Maria Imbaro, Italy; University of Texas Health Science Center at San Antonio, United States of America

## Abstract

**Aims:**

Beta-cell dysfunction is an early event in the natural history of type 2 diabetes. However, its progression is variable and potentially influenced by several clinical factors. We report the baseline data of the BetaDecline study, an Italian prospective multicenter study on clinical predictors of beta-cell dysfunction in type 2 diabetes.

**Materials and Methods:**

Clinical, lifestyle, and laboratory data, including circulating levels of inflammatory markers and non-esterified fatty acids, were collected in 507 type 2 diabetic outpatients on stable treatment with oral hypoglycemic drugs or diet for more than 1 year. Beta-cell dysfunction was evaluated by calculating the proinsulin/insulin ratio (P/I).

**Results:**

At baseline, the subjects in the upper PI/I ratio quartile were more likely to be men and receiving secretagogue drugs; they also showed a borderline longer diabetes duration (P = 0.06) and higher serum levels of glycated hemoglobin (HbA_1c_), fasting blood glucose, and triglycerides. An inverse trend across all PI/I quartiles was noted for BMI and serum levels of total cholesterol (T-C), LDL-C, HDL-C and C reactive protein (CRP), and with homeostatic model assessment (HOMA-B) and HOMA of insulin resistance (HOMA-IR) values (P<0.05 for all). At multivariate analysis, the risk of having a P/I ratio in the upper quartile was higher in the subjects on secretagogue drugs (odds ratio [OR] 4.2; 95% confidence interval [CI], 2.6–6.9) and in the males (OR 1.8; 95% CI, 1.1–2.9).

**Conclusions:**

In the BetaDecline study population, baseline higher PI/I values, a marker of beta-cell dysfunction, were more frequent in men and in patients on secretagogues drugs. Follow-up of this cohort will allow the identification of clinical predictors of beta-cell failure in type 2 diabetic outpatients.

## Introduction

The prevalence of type 2 diabetes mellitus (T2DM) is increasing worldwide [1]. T2DM is sustained by insulin resistance and impaired insulin secretion. Impaired insulin secretion due to either beta-cell dysfunction and/or beta-cell loss is now recognized in the pathogenesis and progression of diabetes. The loss of beta-cell mass and the progressive decline in beta-cell function is an early feature of the natural history of diabetes and it is detectable prior to diagnosis [2]. The United Kingdom Prospective Diabetes Study (UKPDS) showed that beta-cell function, as evaluated by the homeostatic model assessment (HOMA-B) index, was already decreased by 50% by the time of the diagnosis and that it continued to decline over the 6-year observation period, even with on-going hypoglycemic therapy [3].

The relative increase of α-cells mass, another typical defect on Langerhans islets of diabetic subjects, may even precede beta-cells loss, being already observed in normoglycaemic baboons with different degrees of obesity [4].

Beta-cell mass is influenced by a balance between proliferative and pro-apoptotic signals, which may be modulated by various growth factors, cytokines, and hormones, whose specific role in the rate of beta-cell decline remains unclear.

High levels of glucose and free fatty acids (gluco-and lipotoxicity), islet amyloid polypeptide deposition, and circulating inflammatory cytokines have been all implicated in beta-cell apoptosis [4]–[15] Thus, *in vitro* studies have demonstrated that high glucose levels induce an over-expression of pro-apoptotic genes of the Bcl family in cultured pancreatic Islets of Langerhans [8], and the important role of islet amyloid (IA) in the pathogenesis of beta cell dysfunction has been demonstrated in rodent models, non human primates, as well as in human studies [9], [10], [11], [12]; also the influence of inflammation on beta cell function and peripheral insulin resistance has been increasingly recognized [13], [14], [15]

At any rate, whenever it appears, impaired beta-cell function leads to the progressive failure of islet cells to secrete sufficient amounts of insulin to overcome peripheral insulin resistance, ultimately resulting in failure to maintain normal glucose homeostasis over time. However, the rate of beta-cell failure is unpredictable and not all persons with T2DM will need insulin therapy to maintain their blood glucose levels.

Several factors may have a bearing on beta-cell function, including lifestyle, clinical, and metabolic factors, as well as ongoing therapies. The early identification of these clinical predictors could provide clues to developing strategies to ameliorate the long-term management of T2DM.

Here, we report the baseline data of the BetaDecline Study, that will prospectively evaluate the principal clinical and biochemical predictors of beta-cell dysfunction in a cohort of outpatients with T2DM, on stable treatment with oral hypoglycemic agents (OHAs) or diet only, over a 4-year follow-up period.

## Methods

### Subjects

BetaDecline is a prospective, longitudinal multicenter study with the aim to evaluate the rate and clinical predictors of beta-cell decline in T2DM over time. The study population was 507 T2DM outpatients regularly attending nine diabetes care centers. At each center, the fifth eligible patient seen per day was enrolled in the study, after giving written informed consent to participate in the study.

Data were collected from retrospective chart review, laboratory analysis of blood samples taken during visits to a diabetes center as part of routine care, and responses to a questionnaire. At the first and last visits, after 4 years of follow-up, fasting blood samples were taken. At the intermediate visit (after 2 years) only clinical data were collected.

Inclusion criteria were: a physician's diagnosis of T2DM by American Diabetes Association criteria [16]stable treatment with OHAs or diet for ≥1 year; age >40 years at enrolment; continuous care at the clinic (at least 2 visits) for at least 1 year; medical records with a minimum core data set; and completed consent form. All patients meeting these inclusion criteria were considered eligible for participation in the study.

Exclusion criteria were: type 1 diabetes; pregnancy; inability to understand the study purposes or fill out the consent form; participation in a clinical trial in the previous 3 years; current use of insulin or incretin-based therapies (DPP4 inhibitors and/or GLP1 receptor agonists).

All patients were on stable treatment for ≥1 year with dietary therapy and/or on an OHA (metformin, sulfonylureas, glinides, glytazones, acarbose), alone or in combination; none were receiving incretin-based or insulin therapy during the course of the study.

### Ethics statements

Written informed consent was obtained from all participants; the study was conducted in accordance with the Declaration of Helsinki and approved by the local ethics committees:Ethics Committee of the University Hospital Saint Luigi Gonzaga of Orbassano, Turin.

Committee entered in the Register of the Regional Ethics Committees with the order number 7 in the section on committees set up under 'Article 5 of DPGR 16/11/2001 Piedmont region and D.G.R. n. 4/12/2006 78-4807 of operating in compliance with the Decree 24/06/2003 n. 211 and Good Clinical Practice. The Ethics Committee approved the study on 30/06/2008.

### Measurements

Standardized questionnaires were administered to collect lifestyle and clinical data. Potential risk factors for beta-cell dysfunction were classified as follows: co-morbidities - dyslipidemia, hypertension, cardiac disease, long-term micro- and macrovascular complications of diabetes; treatment variables - current antidiabetic drug treatments (name, starting date, dosage), history of antidiabetic drug treatment, antihypertensive therapy, continuous aspirin treatment, statin therapy.

BMI and blood pressure (BP) were measured according to standard procedures. Hypertension was defined as a systolic and/or diastolic BP value ≥130/80 mm Hg, and/or current use of antihypertensive medications. Use of hypolipidemic drugs and/or aspirin was also recorded.

Alcohol consumption was recorded as grams of alcohol/day. Subjects were defined as non-smokers or current smokers; those who had quit within 1 year prior to entry in the study were defined as current smokers.

### Metabolic parameters

At baseline and final visits, blood samples after 12–14 hour fasting were drawn for the determination of blood chemistry parameters, which was performed centrally. After collection, plasma and serum were separated and the aliquots were stored at −20°C until analysis. Fasting blood glucose (FBG) was measured using the glucose oxidase-peroxidase (GOD-POD) method (normal laboratory reference range, 65–110 mg/dL).

Total serum cholesterol, high-density lipoprotein cholesterol (HDL-C), triglycerides, and creatinine levels were measured using standard automated laboratory methods (Roche Diagnostics, Milan, Italy). Low-density lipoprotein cholesterol (LDL-C) concentration was calculated by the Friedwald formula [17].

Glycated hemoglobin (HbA_1c_) was measured on an automated high-performance liquid chromatography (HPLC) analyzer (normal laboratory reference range, 4–6%). Fasting insulin concentration was measured using an automated microparticle enzyme immunosorbent assay (MEIA) (normal reference range, 5–23 MU/L). Plasma proinsulin levels were measured using an enzyme-linked immunosorbent assay (ELISA) (normal reference values, >5.10 pmol/L), and the fasting proinsulin to insulin ratio (PI/I) was calculated. Insulin secretion was assessed with the HOMA-B model and insulin resistance was calculated using the HOMA-IR model [18], [19].

Serum C-reactive protein (CRP) was measured using a turbidimetric assay (reference values, <3.0 mg/L), interleukin 6 (IL-6) by an enzymatic method using Acyl-CoA oxidase (ACS-ACOD-POD) (reference range, 0.0–2.3 ng/L), fasting non-esterified fatty acids (NEFA) by an enzymatic assay (reference range, 0.28–0.89 mmol/L), (CV % and %, respectively). The mean values for self-monitored 2-h post-prandial glucose (2-h PPG) were also recorded.

### Statistical analysis

Data are given as mean ± SD or crude numbers and percentages. Patient characteristics according to gender were compared using unpaired Student's t-test or Mann-Whitney U-test or χ^2^ test. Patient characteristics according to PI/I ratio quartiles were compared using ANOVA for linear trend (normally distributed variables), Kruskall-Wallis one-way ANOVA (continuous variables with non-normal distribution) or χ^2^ test for linear trend (categorical variables). Bivariate associations were estimated using Spearman's rank correlation coefficient. Independent correlates of PI/I values in the upper quartile were evaluated using multiple logistic regression analysis with backward variable selection. Results are expressed as odds ratios (OR) with their respective 95% confidence intervals (95% CI). All statistical comparisons are two-tailed, and a P value<0.05 was considered significant. Statistical analysis was performed using the SPSS program, version 11.0 for Windows (SPSS Inc. Chicago, IL).

## Results

### Baseline clinical characteristics

Study subjects (507, 59% men) were overweight or obese (mean BMI 29.2 kg/m^2^; mean waist circumference 102 cm), with acceptable glucose control, despite the relatively long duration of diabetes (**Table 1**). The FBG levels were high but the 2-h post-prandial glucose (PPG) levels were within the recommended targets. BP and lipid profile were close to the target values. Mean serum fasting insulin and proinsulin levels were 10.1 mIU/L and 9.1 pmol/L, respectively. Mean circulating levels of serum CRP, IL-6, and NEFA were within the reference range (**Table 1**).

**Table 1:** Baseline clinical characteristics of BetaDecline study participants.

	Total	Men	Women	*P*-value men *vs.* women
*No.* (%)	507	298 (58.7)	209 (41.3)	
Age (years)	62.8±8.1	62.7±8.1	63.0±8.2	0.96 (NS)
BMI	29.2±4.9	28.4±4.3	30.2±5.5	<0.0001
Waist circumference (cm)	102±11	102±11	101±11	0.27 (NS)
Diabetes duration (years)	8.8±6.9	9.1±6.6	8.4±7.2	0.08 (NS)
HbA1c (%)	7.2±1.1	7.2±1.0	7.3±1.3	0.05
FBG (mg/dl)	164±59	168±60	160±59	0.03
PPG (mg/dl)	149±39	148±38	151±41	0.49 (NS)
Fasting insulin (mIU/L)	10.1±7.0	9.6±7.3	10.8±6.5	0.003
Fasting proinsulin (pmol/L)	9.1±11.3	10.1±13.1	7.8±7.9	0.001
Systolic blood pressure (mm Hg)	133±16	133±16	133±16	0.69 (NS)
Diastolic blood pressure (mm Hg)	78±8	78±8	79±8	0.62 (NS)
Total cholesterol (mg/dL)	177±41	172±37	184±44	0.002
HDL-cholesterol (mg/dL)	49.6±12.9	46.9±12.1	53.3±13.1	<0.0001
LDL-cholesterol (mg/dL)	103±33	99±32	107±34	0.017
Triglycerides (mg/dL)	132±79	134±83	132±74	0.79 (NS)
CRP (mg/L)	2.8±4.1	2.4±4.2	3.5±3.9	<0.0001
IL-6 (ng/L)	1.46±3.32	1.58±4.23	1.29±1.17	0.65 (NS)
NEFA (mmol/L)	0.6±0.3	0.6±0.2	0.6±0.2	0.002
Antihypertensive drugs – *no.* (%)	350 (69.0)	194 (65.1)	156 (74.6)	0.007
Lipid-lowering drugs - *no.* (%)	309 (60.9)	182 (61.1)	127 (60.8)	0.79 (NS)
Aspirin use – *no.* (%)	228 (45.0)	145 (48.7)	83 (39.7)	0.015
Hypoglicemic drugs – *no.* (%)				
Metformin	425 (83.8)	243 (81.5)	182 (87.1)	0.10 (NS)
Sulfonylureas	179 (35.3)	99 (33.2)	80 (38.3)	0.24 (NS)
TZD	50 (9.9)	29 (9.7)	21 (10.0)	0.91 (NS)
Glinides	81 (16.0)	53 (17.8)	28 (13.4)	0.18 (NS)
Acarbose	11 (2.2)	7 (2.3)	4 (1.9)	1.0 (NS)
Diet only	28 (5.5)	18 (6.0)	10 (4.8)	0.54 (NS)
Current tobacco use – *no.* (%)	90 (17.8)	77 (26.0)	13 (6.2)	<0.0001
Regular alcohol consumption - *no.* (%)	101 (20.0)	89 (30)	12 (5.7)	<0.0001
Physical activity (%)				0.002
No/Occasionally	347 (69.1)	190 (64.9)	157 (75.1)	
<3 times per week	57 (11.4)	40 (13.7)	17 (8.1)	
3–5 times per week	67 (13.3)	39 (13.3)	28 (13.4)	
>5 times per week	28 (5.6)	21 (7.2)	7 (3.3)	

Data are no. (%) and means ± SD. FBG, fasting blood glucose; PPG, postprandial blood glucose; CRP, C-reactive protein; IL-6, interleukin-6; NEFA, non-esterified fatty acids; PI, proinsulin; TZD thiazolidinediones.

During the course of the study, 69% of the subjects were on antihypertensive drugs, 61% on lipid-lowering medications (either statins or fibrates), and 45% were regularly taking aspirin. The majority (84%) was on stable treatment with metformin alone or in combination with other OHAs, 51% were on secretagogues (sulfonylureas and glinides), and only 5% on diet therapy alone.

Questionnaire response to the items investigating lifestyle showed that 18% were current smokers, 20% reported habitual alcohol consumption, and 69% were sedentary, never or only occasionally engaging in physical activity (**Table 1**).

Comparison of baseline data of men versus women (**Table 1**) showed that the women were more often obese (BMI, P<0.0001) and had significantly higher serum levels of HbA1c (P = 0.05), fasting insulin (P = 0.003), total cholesterol (P = 0.002), HDL-C (P<0.0001) and LDL-C (P = 0.017), CRP (P<0.0001), and NEFA (P = 0.002), whereas FBG and fasting proinsulin values were higher in the men (P = 0.03 and P = 0.001, respectively).

The men reported a lower use of antihypertensive medications and a higher use of aspirin; no differences between the sexes were noted for use of lipid-lowering drugs or current treatment with hypoglycemic medications. Tobacco and alcohol use (both P<0.0001) was more frequent among the men; regular physical activity was lower among the women (P = 0.002).

### Baseline clinical characteristics according to Proinsulin/Insulin ratio values

**Figure 1:**
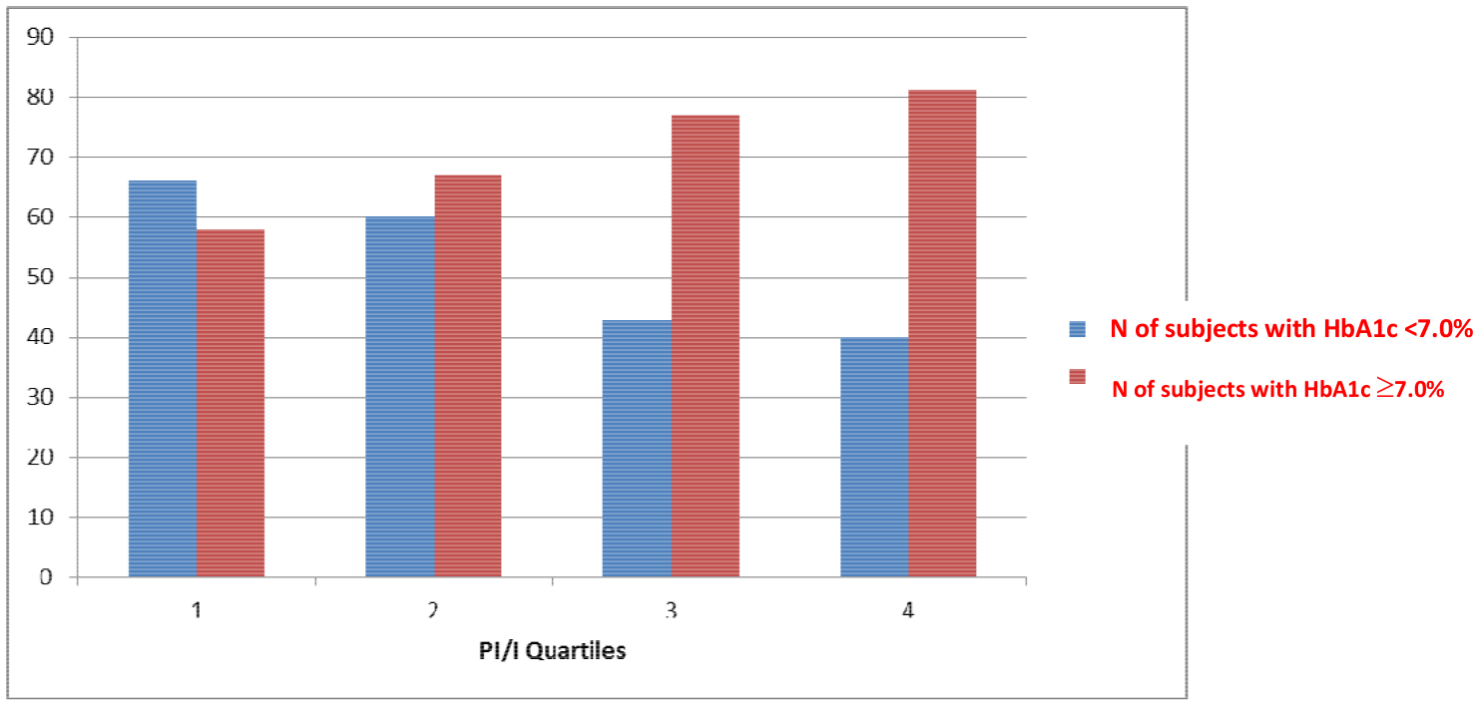
Type 2 diabetic subjects with HbA1c≥7.0% or <7.0% according to the degree of beta-cell dysfunction.

Beta-cell dysfunction was estimated by the the proinsulin/insulin ratio (PI/I); HOMA-B and the degree of insulin resistance (HOMA-IR) were also evaluated. When the study subjects characteristics were stratified according to quartiles of the PI/I ratio, almost 70% of those in the upper PI/I quartile, indicating the highest degree of beta-cell dysfunction, were males. Overall, a higher degree of beta-cell dysfunction was also associated with poorer glucose control; thus, as shown in **Figure 1**, the number of subjects with out-of-target HbA1c values increased with increasing PI/I quartiles (HbA_1c_ ≥7.0%; chi square for linear trend P<0.0001).

To evaluate which factors were associated with beta-cell dysfunction, we stratified the clinical and laboratory characteristics according to PI/I quartiles (**Table 2**); because of the higher prevalence of male subjects among those with the highest PI/I quartile, p-values for comparisons in men and women separately are also presented (**Table 2**).

**Table 2:** Patient characteristics according to quartiles of proinsulin/insulin (PI/I) ratio *in total population and by gender*.

	PI/I ratio quartiles						
Variables	I° ≤0.485	II° 0.486–0.80	III° 0.81–1.355	IV° >1.355	P	P-value in men	P-value in women
Males (%)	37.9	60.8	67.2	68.5	<0.0001		
Age (years)	62.9±8.1	62.4±7.6	62.4±8.7	63.7±8.2	0.51 (NS)	0.90 (NS)	0.21 (NS)
Current smokers (%)	12.1	21.6	22.3	16.3	0.02	0.44 (NS)	0.08 (NS)
Diabetes duration (years)	8.0±6.5	9.4±8.3	8.1±5.7	9.7±6.7	0.06 (NS)	0.14 (NS)	0.58 (NS)
BMI (kg/m^2^)	30.5±5.2	29.5±5.4	28.3±4.2	28.3±4.5	<0.0001	0.04	0.13 (NS)
Waist circumference (cm)	102.7±10.0	101.6±13.3	102.1±10.6	99.4±10.0	0.06 (NS)	0.09 (NS)	0.26 (NS)
HbA1c (%)	7.1±1.1	7.2±1.2	7.3±1.2	7.4±0.9	0.004	0.008	0.04
FBG (mg/dL)	149±54	156±53	173±59	181±66	<0.0001	<0.0001	0.001
PPG (mg/dL)	149±37	152±53	148±31	148±36	0.83 (NS)	0.67 (NS)	0.52 (NS)
SBP (mm Hg)	133±15	132±16	133±16	133±16	0.78 (NS)	0.95 (NS)	1.0 (NS)
DBP (mm Hg)	78.4±8.2	78.4±8.0	78.5±8.0	78.8±9.2	0.68 (NS)	0.71 (NS)	0.95 (NS)
Total cholesterol (mg/dL)	184±42	177±45	173±39	172±36	0.02	0.89 (NS)	0.18 (NS)
HDL-cholesterol (mg/dL)	52.8±13.6	48.6±13.4	47.1±11.0	49.1±12.3	0.007	0.09 (NS)	0.08 (NS)
LDL-cholesterol (mg/dL)	109±32	103±37	99±31	98±29	0.01	0.89 (NS)	0.34 (NS)
Triglycerides (mg/dL)	126±78	135±76	134±76	137±88	0.01	0.34 (NS)	0.48 (NS)
CRP (mg/L)	3.4±3.7	2.7±4.1	3.0±5.2	2.3±3.3	0.001	0.03	0.16 (NS)
IL-6 (ng/L)	1.3±1.0	2.2±6.4	1.3±0.8	1.1±0.9	0.28 (NS)	0.26 (NS)	0.06 (NS)
NEFA (mmol/L)	0.58±0.26	0.58±0.24	0.58±0.25	0.55±0.25	0.64 (NS)	0.57 (NS)	0.31 (NS)
Fasting insulin (mIU/L)	12.7±9.1	11.0±6.5	9.3±5.8	7.4±5.0	<0.0001	<0.0001	0.001
PI (pmol/L)	3.8±2.5	7.0±4.3	9.8±6.4	16.7±19.0	<0.0001	<0.0001	<0.0001
HOMA-B	73.4±63.8	51.6±33.2	37.7±30.4	29.9±33.4	<0.0001	<0.0001	<0.0001
HOMA-IR	4.8±4.4	4.4±4.1	4.0±3.1	3.4±2.7	<0.0001	0.001	0.003
Diet alone (%)	8.1	3.9	7.4	2.4	0.14 (NS)	0.16 (NS)	0.05
Metformin (%)	84.7	85.0	83.6	83.1	0.67 (NS)	0.91 (NS)	0.77 (NS)
Sulfonylureas (%)	29.0	26.0	37.7	47.6	<0.0001	0.003	0.01
Glinides (%)	10.5	11.8	15.6	26.6	<0.0001	0.03	0.01
Any secretagogue (%)	37.9	37.0	53.3	71.0	<0.0001	<0.0001	<0.0001
TZDs (%)	8.9	9.4	13.1	8.1	0.92 (NS)	0.88 (NS)	0.72 (NS)
Acarbose (%)	0.8	1.6	4.1	2.4	0.21 (NS)	0.07 (NS)	0.72 (NS)
Antihypertensives (%)	76.5	71.0	70.1	72.6	0.49 (NS)	0.11 (NS)	0.65 (NS)
Lipid-lowering drugs (%)	59.0	70.4	64.1	60.9	0.97 (NS)	0.07 (NS)	0.38 (NS)
Aspirin (%)	39.8	47.2	44.7	58.5	0.03	0.35 (NS)	0.99 (NS)

Data are no. (%) and means ± SD. FBG, fasting blood glucose; PPG, postprandial blood glucose; SBP and DBP, systolic and diastolic blood pressure; CRP, C-reactive protein; IL-6, interleukin-6; NEFA, non-esterified fatty acids; PI, proinsulin; TZD thiazolidinediones.

In the whole study population, the subjects in the highest PI/I ratio quartile (those with the highest degree of beta-cell dysfunction) were more likely to be smokers and to have a longer duration of diabetes than those in the lower quartiles (P = 0.02 and P = 0.06, respectively). Furthermore, the subjects in the upper PI/I quartile had a lower BMI (P<0.0001) and waist circumference (P<0.06), poorer metabolic control, evaluated as both HbA_1c_ (P<0.004) and FBG values (P<0.0001), without any significant difference in PPG levels. Those in the upper quartile also showed differences in lipid profile, with lower total cholesterol (P = 0.02), LDL-C (P = 0.01), and HDL-C concentrations (P = 0.007), and higher serum triglycerides levels (P = 0.01) (**Table 2**). The CRP values were significantly lower in the subjects in the upper PI/I quartile (P = 0.001), whereas no significant differences in IL-6 or NEFA or systolic and diastolic blood pressure values were noted according to the PI/I ratio in the whole study population. The insulin-sensitivity (HOMA-IR) and secretion indexes (HOMA-B) were both significantly lower in the highest PI/I quartile (P<0.0001).

Among glucose-lowering drugs, the use of secretagogues (sulfonylureas and glinides) was significantly more frequent among subjects with the worst baseline insulin secretory capacity (P<0.0001); conversely, no significant differences in the use of other drugs according to PI/I values were noted. Similarly, no differences were noted for use of antihypertensive and lipid-lowering drugs, whereas a larger number of subjects on aspirin were found in the highest PI/I quartile (P = 0.03).

When men and women were analyzed separately (Table 2), metabolic control as evaluated by FBG and HbA1c levels, as well as fasting insulin, proinsulin, HOMA-B, and HOMA-IR values, the use of sulphonilureas, glinides and overall secretagogues showed the same significant trend across PI/I quartiles observed in the whole population, without any gender-difference (**see File S1**). HDL-C levels were related with PI/I ratio with a borderline significance in both men and women, whereas, waist circumference, BMI, CRP levels significantly decreased across PI/I quartiles only in males. In women, when comparing the lowest with the highest PI/I quartile, higher PI/I values were also associated with slightly lower IL-6 concentrations (1.1 ng/L vs. 1.3 ng/L) and a lower percentage of subjects on diet only (2.4% vs. 8.1% in the highest vs. the lowest PI/I ratio quartile, respectively).

### Factors associated with beta-cell dysfunction at univariate and multivariate analyses

At univariate correlation analysis (**Table 3**), the PI/I ratio negatively correlated with waist circumference, BMI,, CRP,, and positively with HbA_1c_ and FBG,. Significant correlations were also noted for FBG in both sexes when the men and women were considered separately, whereas the inverse correlation with waist circumference and CRP remained significant only for the men.

**Table 3:** Significant univariate correlations (Pearson's test) of proinsulin/insulin (PI/I) ratio with study variables according to sex.

	Total		Men		Women	
	*r*	*P*	*r*	*P*	*r*	*P*
Waist circumference	−0.11	0.02	−0.16	0.01	−0.12	0.097(NS)
BMI	−0.18	<0.0001	−0.13	0.02	−0.17	0.02
HbA_1c_	0.16	<0.0001	0.20	0.001	0.15	0.03
FBG	0.24	<0.0001	0.26	<0.0001	0.20	0.004
CRP	−0.17	<0.0001	−0.16	0.008	−0.15	0.22(NS)

FBG, fasting blood glucose; CRP, C-reactive protein.

At multivariate analysis, when age, gender, BMI, HbA_1c_, total cholesterol, HDL-C, LDL-C, triglycerides, diabetes duration, CRP, IL-6, NEFA, metformin, secretagogues, thiazolidinediones (TZDs) were included in the model, male gender was associated with a 1.8-fold (OR 1.8; 95% CI, 1.1–2.9) higher probability of having beta-cell dysfunction (a PI/I ratio in the upper quartile), whereas the use of secretagogues was associated with a more than 4-fold higher risk (OR 4.20; 95% CI, 2.55–6.91). All other baseline clinical characteristics, including BMI, waist circumference, diabetes duration, metabolic control, gluco- and lipotoxicity measures, and inflammatory markers were not independently associated with beta-cell dysfunction in this population.

## Discussion

T2DM is characterized by insulin resistance and various degrees of beta-cell impairment. Several lines of evidence strongly suggest that preserving beta-cell function is critical to prevent loss of glucose control and progression from prediabetes to overt diabetes [20], [21], [22] or from use of OHAs to insulin therapy in subjects with long-standing disease [3], [23], [24], [25].Nonetheless, insulin therapy is not an inexorable end, and the rate of progression in T2DM appears to differ individually. Because such unpredictability may reflect individual variability in the rate of beta-cell decline, identifying the clinical factors that can predict it holds importance for diabetes management.

In this analysis of baseline data from the BetaDecline study, we examined the degree of beta-cell dysfunction and its potential associations with demographic, clinical, treatment variables, and markers of inflammation and lipotoxicity in a large cohort of T2DM outpatients on stable treatment with diet and/or OHAs for more than 1 year.

Determining beta-cell failure is problematic in clinical practice, as there is no convenient and reliable way to quantify it. Furthermore, beta-cell decline may ensue from an impairment of their mass or function, and these two aspects may not necessarily coincide [19], [26] Several static and dynamic methods are today available to estimate beta-cells functionality, such as HOMA-B, the acute insulin response 5 min after an i.v. glucose load (AIRg), the ratio between the increment in plasma insulin and plasma glucose 30 min after an OGTT (insulinogenic index, ΔI/ΔG-30), as well as more complex methods evaluating the response of plasma insulin to mixed meals during the day or the deconvolution of plasma C-peptide curve using kinetic parameters [24], [27], [28]. Although some of these methods are considered more reliable for the evaluation of beta-cell secretion, most of them are not easily applicable in the clinical practice. In the BetaDecline, beta-cell dysfunction was evaluated by calculating the PI/I ratio, which estimates the capacity of beta cells to convert proinsulin to insulin and may represent an acceptable method to indicate the degree of beta-cell secretion [19], whereas the role of the HOMA-B index, although still used in epidemiological studies, has been largely questioned [19], [28].

In our cohort, the PI/I values were generally within the diabetic range and roughly double than those reported in normo-glycemic subjects [29], [30], [31],confirming the 2–3 fold increase in PI/I values usually observed in diabetic individuals [19], [28].

The first finding of our study is that up to 70% of subjects in the upper quartile of PI/I ratio (those with a greater deficit in beta-cell secretion) were men. This gender-related difference merits deeper analysis since previous experimental and epidemiological studies indicate that women may be partially protected against beta-cell failure, probably thanks to the estrogens influence. Thus, metabolic diseases are less prevalent among premenopausal women [32] and hormone-related differences in regional fat distribution and function obviously play a crucial role in this context [33] Furthermore, two hormonal intervention studies, the Women's Health Initiative Study [34] and the Heart and Oestrogen/Progestin Replacement Study [35], both found a significant reduction in diabetes incidence in subjects receiving estrogens. Convincing evidence also comes from experimental models of insulin-deficient diabetes, where the frequency of diabetes was consistently higher in the male than female animals [36], [37]. The hypothesis that estrogens may be beneficial for pancreatic beta cells has been confirmed by several *in vitro* studies showing that estrogens may protect pancreatic beta cells against apoptosis and prevent insulin-deficient diabetes mellitus through an estrogen receptor-mediated signal or indirectly through the modulation of other hormones [36], [37], [38].

Although estrogen levels and menopausal status were not specifically measured in our study, the observed gender -differences in the degree of beta-cell dysfunction seem to be in line with these reports. Furthermore, since the mean age of the women participating in our study was ∼60 years, with most of them probably post-menopausal, our data also suggest that this relative protection may be extended after menopause. However, the effects of natural menopause on insulin secretion in humans have been poorly studied [36], [39], and the results obtained so far are difficult to interpret because of the complex hormonal and metabolic milieu following menopause and the confounding effect of ageing.Another finding of our study is that the PI/I ratio values are related to the type of ongoing hypoglycemic therapy. Although the cross-sectional nature of our analysis did not allow us to establish causal relationships, our baseline data indicate that the use of secretagogues, either sulfonylureas or glinides, was 4-fold more frequent in subjects having a PI/I ratio in the upper quartile. Several reasons may underline this association, including an indication bias, owing to diabetologists' preferential use of secretagogues in patients with already reduced beta-cell secretion, i.e. in those who are not in good metabolic control. An alternative interpretation could be that these drugs may be associated with baseline higher PI/I ratio also because of their specific mechanism of action [40], [41]. Thus, by attaching to their specific receptor on beta-cells, secretagogues might stimulate not only insulin release, but also its production, therefore leading to an increased production of proinsulin.

Although only the follow-up analysis of our cohort will allow definitive conclusions, similarly to our baseline results, the ADOPT study [42], after initial 6-months increase in the insulinogenic index, demonstrated a faster rate of decline in glyburide treated patients versus those on rosiglitazone or metformin. The initial change with glyburide has been attributed to the alleviation of gluco-toxicity.

Unfortunately, comparative data for the effect of commonly used hypoglycemic drugs on the rate of beta-cell decline are still insufficient [24], [25], [43]. On the other hand, the UKPDS study reported that the progressive worsening of glucose control over time was not influenced by the type of ongoing therapy [3], and consistent with this observation was the finding that other hypoglycemic and/or antihypertensive, lipid-lowering medications and aspirin use were unrelated to PI/I values in our analysis.

Our data also indicate that a higher PI/I ratio is associated with higher HbA_1c_ levels, confirming the major role of beta-cell dysfunction in the deterioration of glucose control.

However, these associations were no longer significant at multivariate analysis, indicating that other factors may have influenced glucose control in our cohort. Notably, when we compared the baseline data of the men versus the women, we found significantly higher serum HbA_1c_ levels among the women, a finding shared by a recently large Italian survey [44] demonstrating that diabetic women are more likely than men to have poorer glycemic control and elevated HbA_1c_ values. These results seem to partly contrast with the finding of fewer women in the group with higher PI/I values (those with the worst glycemic control). However, in the Italian survey [44], as well as in other reports [45] comparative data on beta-cell function in men and women with diabetes were lacking, and the reported gender-related differences in metabolic control were mainly attributed to a poorer quality of diabetes care in women than in men [44], [45].

Our data also showed decreasing values of HOMA-IR across increasing PI/I ratio quartiles, similarly to other studies reporting different degrees of beta-cell dysfunction according to the level of insulin sensibility [20], [21], [22]. Furthermore, features of the metabolic syndrome, including degrees of obesity, showed an overall inverse trend across increasing quartiles of the PI/I ratio, paralleling that of HOMA-IR, indicating that extreme insulin resistance and insulin secretory deficit may depict different phenotypes.

Because insulin resistance is known to precede beta-cell dysfunction in the natural history of T2DM, stratifying individuals on the basis of insulin resistance and insulin secretion measures would ideally identify subjects at different stages of the disease. Subjects with higher baseline PI/I values had a longer diabetes duration and higher HbA1c values, suggesting as one would expect, that with a longer diabetes duration, beta cell function would decline and glucose control deteriorates. However our data indicate that such stratification is not feasible in clinical practice. And although both insulin sensitivity and insulin secretion tend to decline with ageing [30], [46], in our cohort, ageing and duration of diabetes were not independently related to PI/I ratio. The relatively younger age of our study subjects and the lack of reliability of diabetes duration estimates may be likely explanations for these results. The finding that age and diabetes duration were partly unrelated to PI/I values may also indicate that the decline in beta-cell function does not follow a regular timeline but it is variable and potentially influenced by different individual factors instead.

Among such factors, the level of gluco- and lipotoxicity and/or subclinical inflammation may play a role. Added to these, several growth factors, cytokines, hormones, including free fatty acids and inflammatory markers may induce beta-cell apoptosis [4]–[15]. In our study, circulating levels of IL-6, CRP, and NEFA were not related to beta-cell dysfunction at multivariate analysis, and blood sample collection in the fasting state may also have played a role, since circulating NEFA levels may be elevated in the post-absorptive period in diabetic subjects.

Furthermore, there was an overall decreasing trend for circulating levels of these inflammatory and lipotoxicity markers with increasing PI/I ratio values, although with gender-specific associations, which is likely explained by the stronger association of central obesity and chronic subclinical inflammation with the insulin-resistant phenotype. The different strength of the association of PI/I values with obesity and CRP levels in men and with IL-6 in women is difficult to be interpreted and would be likely clarified at the follow-up analysis.

Also, the proportion of smokers was higher in subjects in the top PI/I quartiles, but this association was no longer confirmed at multivariate analysis.

This study has several limitations principally related to its cross-sectional nature. Furthermore, other factors such as lifestyle measures [47] and genetic variants [48]potentially associated with beta-cell decline were not specifically assessed in the current analysis. Another limitation of our study is that we did not use more sophisticated and precise measures of insulin secretion. At this regard, it is important to note that PI/I ratio is a static measure, and its association with male sex and the use of secretagogues in a cross sectional study does not mean that these factors are “causally” related with the decline of beta cell function over time.

However, the aim of the BetaDecline study was to individuate tools to deal with the progressive decline of beta-cell secretion and its potential determinants in the clinical practice, and measuring PI/I ratio together with clinical information may be suitable to this purpose. At this regard, a strong point of the study is that the data were collected in a large outpatient population and so very likely reflect the real-world characteristics of diabetic patients attending a diabetes care center.

In conclusion, in this large cohort of type 2 diabetic outpatients, baseline higher PI/I values, a marker of beta-cell dysfunction, were more frequent in men and those using secretagogues.

These results need to be confirmed in the ongoing follow-up analysis of our study, since identifying specific metabolic and personal variables able to predict the degree of beta-cell loss over time will provide new tools to design better strategies for diabetes prevention and management.

## Supporting Information

File S1 **File containing Tables S1 and S2.** **Table S1**: Patient characteristics according to quartiles of beta-cell insulin secretion, as evaluated by proinsulin/insulin (PI/I) ratio, **i**n male participants. Data are no. (%) and means ± SD. FBG, fasting blood glucose; PPG, postprandial blood glucose; SBP and DBP, systolic and diastolic blood pressure; CRP, C-reactive protein; IL-6, interleukin-6; NEFA, non-esterified fatty acids; PI, proinsulin; TZD thiazolidinediones. **Table S2**: Patient characteristics according to quartiles of beta-cell insulin secretion, as evaluated by proinsulin/insulin (PI/I) ratio, in female participants. Data are no. (%) and means ± SD. FBG, fasting blood glucose; PPG, postprandial blood glucose; SBP and DBP, systolic and diastolic blood pressure; CRP, C-reactive protein; IL-6, interleukin-6; NEFA, non-esterified fatty acids; PI, proinsulin; TZD thiazolidinediones.
(DOC)

## References

[1] Mokdad AH, Ford ES, Bowman BA, Dietz WH, Vinicor F, et al (2001) Prevalence of obesity, diabetes, and obesity-related health risk factors. JAMA 289: 76–9. 10.1001/jama.289.1.76 12503980

[2] Kahn SE (2003) The relative contributions of insulin resistance and beta-cell dysfunction to the pathophysiology of Type 2 diabetes. Diabetologia 46: 3–19. 12637977 10.1007/s00125-002-1009-0

[3] Wright A, Burden AC, Paisey RB, Cull CA, Holman RR (2002) U.K. Prospective Diabetes Study Group. Sulfonylurea inadequacy: efficacy of addition of insulin over 6 years in patients with type 2 diabetes in the U.K. Prospective Diabetes Study (UKPDS 57). Diabetes Care 25: 330–6. 11815505 10.2337/diacare.25.2.330

[4] Guardado-Mendoza R, Jimenez-Ceja L, Majluf-Cruz A, Kamath S, Fiorentino TV, et al (2013) Impact of obesity severity and duration on pancreatic β- and α-cell dynamics in normoglycemic non-human primates. Int J Obes (Lond) 37: 1071–8. 23229736 10.1038/ijo.2012.205 PMC3906680

[5] Rhodes CJ (2005) Type 2 diabetes-a matter of beta-cell life and death? Science 307: 380–384. 15662003 10.1126/science.1104345

[6] Lee SC, Pervaiz S (2007) Apoptosis in the pathophysiology of diabetes mellitus. Int J Biochem Cell Biol 39: 497–504. 17074529 10.1016/j.biocel.2006.09.007

[7] Folli F, Okada T, Perego C, Gunton J, Liew CW, et al (2011) Altered insulin receptor signalling and β-cell cycle dynamics in type 2 diabetes mellitus. PLoS One 6 (11) e28050. 22140505 10.1371/journal.pone.0028050 PMC3227614

[8] Federici M, Hribal M, Perego L, Ranalli M, Caradonna Z, et al (2001) High glucose causes apoptosis in cultured human pancreatic islets of Langerhans: a potential role for regulation of specific Bcl family genes toward an apoptotic cell death program. Diabetes 50: 1290–301. 11375329 10.2337/diabetes.50.6.1290

[9] Verchere CB, D'Alessio DA, Palmiter RD, Weir GC, Bonner-Weir S, et al (1996) Islet amyloid formation associated with hyperglycemia in transgenic mice with pancreatic beta cell expression of human islet amyloid polypeptide. Proc Natl Acad Sci U S A 93: 3492–6. 8622964 10.1073/pnas.93.8.3492 PMC39637

[10] Guardado-Mendoza R, Davalli AM, Chavez AO, Hubbard GB, Dick EJ, et al (2009) Pancreatic islet amyloidosis, beta-cell apoptosis, and alpha-cell proliferationare determinants of islet remodeling in type-2 diabetic baboons. Proc Natl Acad Sci U S A 106: 13992–7. 19666551 10.1073/pnas.0906471106 PMC2729008

[11] Jurgens CA, Toukatly MN, Fligner CL, Udayasankar J, Subramanian SL, et al (2011) β-cell loss and β-cell apoptosis in human type 2 diabetes are related to islet amyloid deposition. Am J Pathol 178: 2632–40. 21641386 10.1016/j.ajpath.2011.02.036 PMC3123989

[12] Westermark P, Andersson A, Westermark GT (2011) Islet amyloid polypeptide, islet amyloid, and diabetes mellitus. Physiol Rev 91: 795–826. 21742788 10.1152/physrev.00042.2009

[13] Federici M, Hribal ML, Menghini R, Kanno H, Marchetti V, et al (2005) Timp3 deficiency in insulin receptor-haploinsufficient mice promotes diabetes and vascular inflammation via increased TNF-alpha. J Clin Invest 115: 3494–505. 16294222 10.1172/JCI26052 PMC1283942

[14] Monroy A, Kamath S, Chavez AO, Centonze VE, Veerasamy M, et al (2009) Impaired regulation of the TNF-alpha converting enzyme/tissue inhibitor of metalloproteinase 3 proteolytic system in skeletal muscle of obese type 2 diabetic patients: a new mechanism of insulin resistance in humans. Diabetologia 52: 2169–81. 19633828 10.1007/s00125-009-1451-3 PMC2845986

[15] Tripathy D, Daniele G, Fiorentino TV, Perez-Cadena Z, Chavez-Velasquez A, et al (2013) Pioglitazone improves glucose metabolism and modulates skeletal muscle TIMP-3-TACE dyad in type 2 diabetes mellitus: a randomised, double-blind, placebo-controlled, mechanistic study Diabetologia. 56: 2153–63. 10.1007/s00125-013-2976-z 23811853

[16] The Expert Committee on the Diagnosis and Classification of Diabetes Mellitus (1997) Report of the Expert Committee on the Diagnosis and Classification of Diabetes Mellitus. Diabetes Care 20: 1183–97. 9203460 10.2337/diacare.20.7.1183

[17] Friedewald WT, Levy RI, Fredrickson DS (1972) Estimation of the concentration of low-density lipoprotein cholesterol in plasma, without use of the preparative ultracentrifuge. Clin Chem 18: 499–502. 4337382

[18] Wallace TM, Levy JC, Matthews DR (2004) Use and abuse of HOMA modeling. Diabetes Care 27 (6) 1487–95. 15161807 10.2337/diacare.27.6.1487

[19] Kahn SE, Carr DB, Faulenbach MV, Utzschneider KM (2008) An examination of beta-cell function measures and their potential use for estimating beta-cell mass. Diabetes Obes Metab 10 Suppl 4: 63–76. 18834434 10.1111/j.1463-1326.2008.00945.x

[20] Kitabchi AE, Temprosa M, Knowler WC, Kahn SE, Fowler SE, et al (2005) Diabetes Prevention Program Research Group. Role of insulin secretion and sensitivity in the evolution of type 2 diabetes in the diabetes prevention program: effects of lifestyle intervention and metformin. Diabetes 54: 2404–14. 16046308 10.2337/diabetes.54.8.2404 PMC1360738

[21] Weyer C, Bogardus C, Mott DM, Pratley RE (1999) The natural history of insulin secretory dysfunction and insulin resistance in the pathogenesis of type 2 diabetes mellitus. J Clin Invest 104: 787–94. 10491414 10.1172/JCI7231 PMC408438

[22] DeFronzo RA, Banerji MA, Bray GA, Buchanan TA, Clement S, et al (2010) ACT NOW Study Group. Determinants of glucose tolerance in impaired glucose tolerance at baseline in the Actos Now for Prevention of Diabetes (ACT NOW) study. Diabetologia 53: 435–45. 20012012 10.1007/s00125-009-1614-2

[23] Kahn SE, Haffner SM, Heise MA, Herman WH, Holman RR, et al (2006) ADOPT Study Group. Glycemic durability of rosiglitazone, metformin, or glyburide monotherapy. N Engl J Med 355: 2427–43. 17145742 10.1056/NEJMoa066224

[24] Bonora E (2008) Protection of pancreatic beta-cells: is it feasible? Nutr Metab Cardiovasc Dis 18: 74–83. 18096375 10.1016/j.numecd.2007.05.004

[25] Mudaliar S (2013) Choice of early treatment regimen and impact on β-cell preservation in type 2 diabetes. Int J Clin Pract 67: 876–87. 23952467 10.1111/ijcp.12154

[26] Brunzell JD, Robertson RP, Lerner RL, Hazzard WR, Ensinck JW, et al (1976) Relationships between fasting plasma glucose levels and insulin secretion during intravenous glucose tolerance tests. J Clin Endocrinol Metab 42: 222–9. 1262429 10.1210/jcem-42-2-222

[27] Phillips DI, Clark PM, Hales CN, Osmond C (1994) Understanding oral glucose tolerance: comparison of glucose or insulin measurements during the oral glucose tolerance test with specific measurements of insulin resistance and insulin secretion. Diabet Med 11: 286–92.. 8033528 10.1111/j.1464-5491.1994.tb00273.x

[28] Reaven GM (2009) Insulin secretory function in type 2 diabetes: Does it matter how you measure it? J Diabetes 1: 142–50. 20923533 10.1111/j.1753-0407.2009.00016.x

[29] Festa A, Williams K, Hanley AJ, Haffner SM (2008) Beta-cell dysfunction in subjects with impaired glucose tolerance and early type 2 diabetes: comparison of surrogate markers with first-phase insulin secretion from an intravenous glucose tolerance test. Diabetes 57: 1638–44. 18332099 10.2337/db07-0954

[30] Bryhni B, Arnesen E, Jenssen TG (2010) Associations of age with serum insulin, proinsulin and the proinsulin-to-insulin ratio: a cross-sectional study. BMC Endocr Disord 10: 21. 21162746 10.1186/1472-6823-10-21 PMC3020169

[31] Røder ME, Dinesen B, Hartling SG, Houssa P, Vestergaard H, et al (1999) Intact proinsulin and beta-cell function in lean and obese subjects with and without type 2 diabetes. Diabetes Care 22: 609–14. 10189540 10.2337/diacare.22.4.609

[32] Ford ES (2005) Prevalence of the metabolic syndrome defined by the International Diabetes Federation among adults in the U.S. Diabetes Care 28: 2745–49. 16249550 10.2337/diacare.28.11.2745

[33] Shi H, Seeley RJ, Clegg DJ (2009) Sexual differences in the control of energy homeostasis. Front Neuroendocrinol 30: 396–404. 19341761 10.1016/j.yfrne.2009.03.004 PMC4517605

[34] Margolis KL, Bonds DE, Rodabough RJ, Tinker L, Phillips LS, et al (2004) Women's Health Initiative Investigators. Effect of oestrogen plus progestin on the incidence of diabetes in postmenopausal women: results from the Women's Health Initiative Hormone Trial. Diabetologia 47: 1175–87. 15252707 10.1007/s00125-004-1448-x

[35] Kanaya AM, Herrington D, Vittinghoff E, Lin F, Grady D, et al (2003) Heart and Estrogen/progestin Replacement Study. Glycemic effects of postmenopausal hormone therapy: the Heart and Estrogen/progestin Replacement Study. A randomized, double-blind, placebo-controlled trial. Ann Intern Med 138: 1–9. 10.7326/0003-4819-138-1-200301070-00005 12513038

[36] Godsland IF (2005) Oestrogens and insulin secretion. Diabetologia 48: 2213–20. 16193292 10.1007/s00125-005-1930-0

[37] Tiano JP, Mauvais-Jarvis F (2012) Importance of oestrogen receptors to preserve functional β-cell mass in diabetes. Nat Rev Endocrinol 8: 342–51. 22330739 10.1038/nrendo.2011.242

[38] Le May C, Chu K, Hu M, Ortega CS, Simpson ER, et al (2006) Estrogens protect pancreatic beta-cells from apoptosis and prevent insulin-deficient diabetes mellitus in mice. Proc Natl Acad Sci U S A 103: 9232–37. 16754860 10.1073/pnas.0602956103 PMC1482595

[39] Chandler-Laney PC, Phadke RP, Granger WM, Muñoz JA, Man CD, et al (2010) Adiposity and β-cell function: relationships differ with ethnicity and age. Obesity 18: 2086–92. 20300083 10.1038/oby.2010.44 PMC3074461

[40] Dworacka M, Abramczyk M, Winiarska H, Kuczynski S, Borowska M, et al (2006) Disproportionately elevated proinsulin levels in type 2 diabetic patients treated with sulfonylurea. Int J Clin Pharmacol Ther 44: 14–21. 16425966 10.5414/cpp44014

[41] Pfützner A, Lorra B, Abdollahnia MR, Kann PH, Mathieu D, et al (2006) The switch from sulfonylurea to preprandial short-acting insulin analog substitution has an immediate and comprehensive beta-cell protective effect in patients with type 2 diabetes mellitus. Diabetes Technol Ther 8: 375–84. 16800759 10.1089/dia.2006.8.375

[42] Kahn SE, Lachin JM, Zinman B, Haffner SM, Aftring RP, et al (2011) ADOPT Study. Effects of rosiglitazone, glyburide, and metformin on β-cell function and insulin sensitivity in ADOPT. Diabetes 60: 1552–60.. 21415383 10.2337/db10-1392 PMC3292330

[43] Pfützner A, Forst T (2011) Elevated intact proinsulin levels are indicative of Beta-cell dysfunction, insulin resistance, and cardiovascular risk: impact of the antidiabetic agent pioglitazone. J Diabetes Sci Technol 5: 784–93. 21722594 10.1177/193229681100500333 PMC3192645

[44] Rossi MC, Cristofaro MR, Gentile S, Lucisano G, Manicardi V, et al (2013) Sex Disparities in the Quality of Diabetes Care: Biological and Cultural Factors May Play a Different Role for Different Outcomes: A cross-sectional observational study from the AMD Annals initiative. Diabetes Care 36: 3162–68. 23835692 10.2337/dc13-0184 PMC3781503

[45] Rivellese AA, Riccardi G, Vaccaro O (2010) Cardiovascular risk in women with diabetes. Nutr Metab Cardiovasc Dis. 20: 474–80. 20621459 10.1016/j.numecd.2010.01.008

[46] Basu R, Breda E, Oberg AL, Powell CC, Dalla Man C, et al (2003) Mechanisms of the age-associated deterioration in glucose tolerance: contribution of alterations in insulin secretion, action, and clearance. Diabetes 52: 1738–48. 12829641 10.2337/diabetes.52.7.1738

[47] De Mello VD, Lindström J, Eriksson J, Ilanne-Parikka P, Keinänen-Kiukaanniemi S, et al (2012) Insulin secretion and its determinants in the progression of impaired glucose tolerance to type 2 diabetes in impaired glucose-tolerant individuals: the Finnish Diabetes Prevention Study. Diabetes Care 35: 211–17. 22210578 10.2337/dc11-1272 PMC3263888

[48] Strawbridge RJ, Dupuis J, Prokopenko I, Barker A, Ahlqvist E, et al (2011) Genome-wide association identifies nine common variants associated with fasting proinsulin levels and provides new insights into the pathophysiology of type 2 diabetes. Diabetes 60: 2624–34.. 21873549 10.2337/db11-0415 PMC3178302

