# Supplementary material for: Factors Associated with Beta-Cell Dysfunction in Type 2 Diabetes: The BETADECLINE Study
Source: PLoS One. 2014 Oct 27;9(10):e109702. doi: 10.1371/journal.pone.0109702 (PMC4210056; doi:10.1371/journal.pone.0109702)
Supplement: File S1 — File containing Tables S1 and S2. Table S1: Patient characteristics according to quartiles of beta-cell insulin secretion, as evaluated by proinsulin/insulin (PI/I) ratio, in male participants. Data are no. (%) and means ± SD. FBG, fasting blood glucose; PPG, postprandial blood glucose; SBP and DBP, systolic and diastolic blood pressure; CRP, C-reactive protein; IL-6, interleukin-6; NEFA, non-esterified fatty acids; PI, proinsulin; TZD thiazolidinediones. Table S2: Patient characteristics according to quartiles of beta-cell insulin secretion, as evaluated by proinsulin/insulin (PI/I) ratio, in female participants. Data are no. (%) and means ± SD. FBG, fasting blood glucose; PPG, postprandial blood glucose; SBP and DBP, systolic and diastolic blood pressure; CRP, C-reactive protein; IL-6, interleukin-6; NEFA, non-esterified fatty acids; PI, proinsulin; TZD thiazolidinediones. (DOC) [file pone.0109702.s001.doc]

**Supplemental Tables**

**Table S1-** Patient characteristics according to quartiles of beta-cell insulin secretion, as evaluated by proinsulin/insulin (PI/I) ratio, **i**n male participants.

| Variable | PI/I ratio quartiles | | | | *P* |
| --- | --- | --- | --- | --- | --- |
|  | I°  ≤0.485 | II°  0.486-0.80 | III°  0.81-1.355 | IV°  >1.355 |  |
|  |  |  |  |  |  |
| Age (years) | 63.2±8.4 | 62.6±7.2 | 62.6±9.2 | 63.0±7.5 | 0.90(NS) |
| Current smokers (%) | 23.4 | 34.2 | 28.4 | 19.0 | 0.44(NS) |
| Diabetes duration (years) | 8.7±6.9 | 9.1±7.1 | 8.1±6.0 | 10.1±6.6 | 0.14(NS) |
| BMI (kg/m^2^) | 29.7±5.1 | 29.2±4.5 | 27.4±3.3 | 28.1±4.2 | 0.04 |
| Waist circumference (cm) | 104.7±10.8 | 103.4±13.3 | 101.5±9.6 | 99.5±9.5 | 0.09(NS) |
| HbA1c (%) | 6.9±1.0 | 7.1±1.1 | 7.2±1.0 | 7.4±1.0 | 0.008 |
| FBG (mg/dL) | 148±54 | 161±57 | 164±49 | 188±70 | <0.0001 |
| PPG (mg/dL) | 145±35 | 146±52 | 143±32 | 153±35 | 0.67(NS) |
| SBP (mm Hg) | 133±15 | 132±17 | 132±14 | 132±17 | 0.95(NS) |
| DBP (mm Hg) | 77.6±8.1 | 78.0±7.9 | 78.1±7.5 | 79.0±9.6 | 0.71(NS) |
| Total cholesterol (mg/dL) | 175±36 | 172±41 | 171±38 | 170±33 | 0.89(NS) |
| HDL-cholesterol (mg/dL) | 50.0±13.7 | 44.6±12.5 | 46.3±10.2 | 47.1±11.5 | 0.09(NS) |
| LDL-cholesterol (mg/dL) | 104±33 | 100±36 | 97±31 | 96±26 | 0.89(NS) |
| Triglycerides (mg/dL) | 114±54 | 144±86 | 133±73 | 138±102 | 0.34(NS) |
| CRP (mg/L) | 2.6±2.6 | 2.4±4.3 | 2.4±5.6 | 2.2±3.3 | 0.03 |
| IL-6 (ng/L) | 1.2±1.0 | 2.8±8.1 | 1.2±0.9 | 1.1±1.0 | 0.26(NS) |
| NEFA (mmol/L) | 0.58±0.26 | 0.53±0.21 | 0.56±0.24 | 0.53±0.26 | 0.57(NS) |
| Fasting insulin (mIU/L) | 13.8±11.6 | 11.4±7.0 | 8.2±5.1 | 7.2±4.9 | <0.0001 |
| PI (pmol/L) | 4.0±3.0 | 7.3±4.7 | 9.1±6.3 | 17.4±21.2 | <0.0001 |
| HOMA-B | 75.3±67.1 | 50.2±32.0 | 35.4±29.3 | 29.1±36.2 | <0.0001 |
| HOMA-IR | 5.3±5.9 | 4.7±4.6 | 3.3±2.2 | 3.4±2.6 | 0.001 |
| Diet alone (%) | 8.5 | 2.6 | 9.8 | 3.5 | 0.16(NS) |
| Metformin (%) | 83.0 | 81.6 | 80.5 | 83.5 | 0.91(NS) |
| Sulfonylureas (%) | 25.5 | 25.0 | 29.3 | 47.1 | 0.003 |
| Glinides (%) | 10.6 | 14.5 | 19.5 | 24.7 | 0.03 |
| Any secretagogue (%) | 34.0 | 38.2 | 48.8 | 68.2 | <0.0001 |
| TZDs (%) | 8.5 | 10.5 | 11.0 | 8.2 | 0.88(NS) |
| Acarbose (%) | 0.0 | 0.0 | 4.9 | 3.5 | 0.07(NS) |
| Antihypertensives (%) | 70.2 | 67.1 | 63.4 | 63.5 | 0.11(NS) |
| Lipid-lowering drugs (%) | 59.6 | 69.7 | 63.4 | 57.1 | 0.07(NS) |
| Aspirin (%) | 39.3 | 51.3 | 46.3 | 56.5 | 0.35(NS) |

Data are no. (%) and means ± SD. FBG, fasting blood glucose; PPG, postprandial blood glucose; SBP and DBP, systolic and diastolic blood pressure; CRP, C-reactive protein; IL-6, interleukin-6; NEFA, non-esterified fatty acids; PI, proinsulin; TZD thiazolidinediones.

**Table S2-** Patient characteristics according to quartiles of beta-cell insulin secretion, as evaluated by proinsulin/insulin (PI/I) ratio, in female participants.

| Variable | PI/I ratio quartiles | | | | *P* |
| --- | --- | --- | --- | --- | --- |
|  | I°  ≤0.485 | II°  0.486-0.80 | III°  0.81-1.355 | IV°  >1.355 |  |
|  |  |  |  |  |  |
| Age (years) | 62.8±8.0 | 62.0±8.2 | 62.0±7.4 | 65.1±9.6 | 0.21(NS) |
| Current smokers (%) | 5.2 | 2.0 | 10.0 | 10.3 | 0.08(NS) |
| Diabetes duration (years) | 7.5±6.3 | 9.9±9.9 | 8.0±5.0 | 8.9±6.9 | 0.58(NS) |
| BMI (kg/m^2^) | 31.0±5.2 | 30.1±6.5 | 30.1±5.1 | 28.8±5.3 | 0.13(NS) |
| Waist circumference (cm) | 101.5±9.7 | 99.2±13.3 | 103.2±12.1 | 99.2±11.5 | 0.26(NS) |
| HbA1c (%) | 7.2±1.7 | 7.2±1.4 | 7.7±1.5 | 7.4±0.8 | 0.04 |
| FBG (mg/dL) | 149±55 | 147±46 | 190±74 | 166±54 | 0.001 |
| PPG (mg/dL) | 151±38 | 160±56 | 155±29 | 138±39 | 0.52(NS) |
| SBP (mm Hg) | 134±16 | 133±14 | 135±19 | 133±14 | 1.0(NS) |
| DBP (mm Hg) | 78.8±8.3 | 79.1±8.3 | 79.3±9.1 | 78.4±8.4 | 0.95(NS) |
| Total cholesterol (mg/dL) | 189±44 | 185±50 | 176±40 | 177±39 | 0.18(NS) |
| HDL-cholesterol (mg/dL) | 54.5±13.3 | 54.6±12.1 | 48.9±12.8 | 53.1±13.1 | 0.08(NS) |
| LDL-cholesterol (mg/dL) | 112±32 | 109±38 | 103±31 | 102±35 | 0.34(NS) |
| Triglycerides (mg/dL) | 133±89 | 123±56 | 135±84 | 135±52 | 0.48(NS) |
| CRP (mg/L) | 3.8±4.2 | 3.3±3.9 | 4.0±4.0 | 2.7±3.2 | 0.16(NS) |
| IL-6 (ng/L) | 1.3±1.0 | 1.4±1.8 | 1.4±0.7 | 1.1±0.8 | 0.06 (NS) |
| NEFA (mmol/L) | 0.59±0.26 | 0.66±0.26 | 0.62±0.26 | 0.60±0.24 | 0.31(NS) |
| Fasting insulin (mIU/L) | 12.1±7.1 | 10.4±5.7 | 11.4±6.6 | 7.7±5.4 | 0.001 |
| PI (pmol/L) | 3.6±2.0 | 6.5±3.8 | 11.2±6.5 | 15.0±12.8 | <0.0001 |
| HOMA-B | 72.2±68.6 | 53.4±35.4 | 42.5±32.3 | 31.6±26.6 | <0.0001 |
| HOMA-IR | 4.5±3.2 | 3.9±3.2 | 5.4±4.0 | 3.3±3.0 | 0.003 |
| Diet alone (%) | 7.8 | 6.1 | 2.5 | 0.0 | 0.05 |
| Metformin (%) | 85.7 | 89.8 | 90.0 | 82.1 | 0.77(NS) |
| Sulfonylureas (%) | 31.2 | 28.6 | 55.0 | 48.7 | 0.01 |
| Glinides (%) | 10.4 | 8.2 | 7.5 | 30.8 | 0.01 |
| Any secretagogue (%) | 40.3 | 36.7 | 62.5 | 76.9 | <0.0001 |
| TZDs (%) | 9.1 | 8.2 | 17.5 | 7.7 | 0.72(NS) |
| Acarbose (%) | 1.3 | 4.1 | 2.5 | 0.0 | 0.72(NS) |
| Antihypertensives (%) | 75.3 | 73.5 | 75.0 | 71.8 | 0.65(NS) |
| Lipid-lowering drugs (%) | 53.2 | 69.4 | 57.5 | 64.1 | 0.38(NS) |
| Aspirin (%) | 37.7 | 36.7 | 32.5 | 53.8 | 0.99(NS) |

Data are no. (%) and means ± SD. FBG, fasting blood glucose; PPG, postprandial blood glucose; SBP and DBP, systolic and diastolic blood pressure; CRP, C-reactive protein; IL-6, interleukin-6; NEFA, non-esterified fatty acids; PI, proinsulin; TZD thiazolidinediones.
